# Depression and quality of life in children with sickle cell disease: the effect of social support

**DOI:** 10.1186/s12888-015-0461-6

**Published:** 2015-04-11

**Authors:** Mohammad Gamal Sehlo, Hayat Zakaria Kamfar

**Affiliations:** Department of Medicine, Psychiatry Unit, Faculty of Medicine, King Abdulaziz University, Jeddah 21589, P. O. Box 80205, Jeddah, Kingdom of Saudi Arabia; Department of Psychiatry, Zagazig University, Zagazig, Egypt; Department of Pediatrics, Faculty of Medicine, King Abdulaziz University, Jeddah, Kingdom of Saudi Arabia

**Keywords:** Depression, Quality of life, Social support, Sickle cell disease

## Abstract

**Background:**

The majority of available studies have shown that children with sickle cell disease (SCD) have a higher risk of depressive symptoms than those without. The present study aimed to: assess the prevalence of depression in a sample of children with SCD; evaluate the association between disease severity, social support and depression, and the combined and/or singular effect on health-related quality of life (HRQL) in children with SCD; and show the predictive value of social support and disease severity on depression.

**Methods:**

A total of 120 children were included in the study, 60 (group I) with SCD and 60 matched, healthy control children (group II). Depression was assessed in both groups using the Children’s Depression Inventory (CDI) and the Children’s Depression Inventory-Parent (CDI-P). Children with CDI and CDI-P scores of more than 12 were interviewed for further assessment of depression using the Diagnostic Interview Schedule for Children Version IV (DISC-IV). The Pediatric Quality of Life Inventory Version 4.0 Generic Core Scales (PedsQL 4.0) was used to assess HRQL in both groups, and social support was measured with the Child and Adolescent Social Support Scale (CASSS).

**Results:**

Eight (13%) of the 60 children with SCD had CDI and CDI-P scores of more than 12 (CDI mean score 14.50 ± 1.19, CDI-P mean score 14.13 ± 1.12), and were diagnosed as having clinical depression using the diagnostic interview DISC-IV. For group I, HRQL was poor across all PedsQL 4.0 domains in both self- and parent-reports (P < 0.001) compared with group II. A higher level of parent support was a significantly associated with decreased depressive symptoms, demonstrated by lower CDI scores. Better quality of life was shown by the associated higher total PedsQL 4.0 self-scores of children with SCD (B = −1.79, P = 0.01 and B = 1.89, P = 0.02 respectively).

**Conclusions:**

The present study demonstrates that higher levels of parent support were significantly associated with decreased depressive symptoms and better quality of life in children with SCD. Interventions focused on increasing parent support may be an important part of treatment for depression in children with SCD.

## Background

Sickle cell disease (SCD) is a group of chronic, recessively inherited blood diseases, of which the most common type is the homozygous SS state. The interaction of sickle hemoglobin with other globin chain abnormalities also results in clinically significant but often milder diseases, for example, SC disease [1]. SCD can result in a host of physiological, cognitive, and psychosocial comorbidities, including chronic and acute anemia, infection, stroke, severe pain episodes, delayed puberty, and academic underachievement [2,3]. Depression is one of the most common complications of chronic illnesses [4,5], and childhood depression is a medical problem that can have serious consequences if it is unrecognized and untreated [6,7]. The majority of available studies have shown that children with SCD have a higher risk of depressive symptoms [8-11], although some studies failed to show significant levels of anxiety and depression [12]. In a recent study, approximately half of the children and adolescents with SCD were diagnosed with either dysthymia (90%) or major depression (10%) [8]. Health-related quality of life (HRQL) in children and adolescents with SCD has also been studied, with the majority of these studies reporting lower HRQL compared to normal controls [13-15]. Researchers have reported that children and adolescents with high perceived levels of social support have often been found to have fewer adjustment problems [16-18]. A study of sickle cell self-help support groups found that group participation reduced feelings of depression and that the longer patients participated in the group, the fewer psychological symptoms they reported [19]. To the knowledge of the present authors, this is the first study that assesses the association between various types of social support and depression and quality of life in children with sickle cell disease. The present study hypothesized that increased social support is associated with decreased depressive symptoms and better quality of life, and aimed to:

1. Assess the prevalence of depression in a sample of children with SCD.
2. Evaluate the association between disease severity, social support, and depression, and the combined and/or singular effect on HRQL in children with SCD.
3. Show the predictive value of social support and disease severity on depression.

## Methods

### Study design

The present study used a case–control design that included 120 children; 60 with SCD, randomly selected (by systematic random sampling) from the outpatient clinic and inpatient Department of Pediatrics at King Abdulaziz University Hospital in Jeddah, Saudi Arabia (group I), and 60 children as a matched, healthy control group (group II). The study was conducted from January 2012 to January 2013. Both groups underwent psychological assessment for comparison. The participating children were interviewed with a parent present. Informed consent was obtained from participants and their parents, and ethical approval was obtained from the local ethical committee of King Abdulaziz University Hospital.

### Participants

The study sample consisted of two groups. Group I comprised 60 children with SCD aged 10–15. Exclusion criteria were: a history of a previous cerebrovascular accident, established by history-taking and reviewing the patient’s medical records; any concomitant serious medical problems (other than those secondary to SCD except cerebrovascular accident), established by physical examination and reviewing the patient’s medical records; any past or family history of psychiatric disorder; and, any childhood disorder such as mental retardation, autism, attention deficit hyperkinetic disorder, and language barrier that may interfere with the patient understanding and answering the interview questions. Group II comprised 60 matched, healthy control children, aged 9–15, without any past or family history of any psychiatric disorder and not receiving any psychotropic medications (established by psychiatric history-taking from the children and their parents). The present study aimed to evaluate the association between disease severity, social support, and depression as well as the combined and/or singular effect on HRQL in children with SCD. Participants’ sociodemographic data were recorded using a questionnaire developed by the study team.

### Measures

#### Evaluation of depression

Depression was measured with: (1) The Children’s Depression Inventory (CDI) [20] which is a child self-report inventory of depressive symptoms. It has high internal consistency, test-retest reliability, and established validity [21], with an alpha coefficient of 0.86. The CDI is the most established and widely used measure of depressive symptoms for children [22,23]. The CDI is a 27-item scale appropriate for children and adolescents aged 7–17 years, and yields scores for five subscales: negative mood, interpersonal problems, ineffectiveness, anhedonia, and negative self-esteem. There are three response options for each item: 0 = absence of symptoms, 1 = moderate symptoms, and 3 = severe symptoms, with higher scores indicating increasing severity. The maximum score is 54, with a score over 12 suggesting a depressive state. The CDI can be used as a screening tool for depressive symptoms in medically ill children [24,25]. In addition, the CDI has been shown to be suitable for use with a mixed sample of in- and out-patients with chronic diseases and acute conditions [25].

(2) The Children’s Depression Inventory-Parent (CDI-P) [26] was used to assess the convergence of parent-report with child-report of depression. The CDI-P is derived directly from the CDI and is scored identically. It has high test-retest reliability (0.75) and internal consistency (0.74), is well correlated with the CDI [27] and has an alpha coefficient of 0.88.

(3) Children with CDI and CDI-P scores of more than 12 were interviewed for further assessment of depression using the Diagnostic Interview Schedule for Children, Version IV (DISC-IV) [28]; a structured psychiatric diagnostic interview for children and adolescents aged 6–18 years, based on DSM IV diagnostic criteria. Although the Diagnostic Interview Schedule for Children (DISC) was designed to be used in large-scale epidemiological studies, it has also been used as an adjunct in public health screening to increase the specificity of brief initial screening [29], and has been used successfully in treatment studies to assess inclusion and exclusion criteria [30]. The DISC offers greater potential for standardization and reduced error [28].

### Evaluation of health-related quality of life

Health-related quality of life (HRQL) was measured with the Pediatric Quality of Life Inventory, Version 4.0 Generic Core Scales (PedsQL 4.0). This is a 23-item scale for children and adolescents aged 8–18 years encompassing: a) physical functioning (8 items); b) emotional functioning (5 items); c) social functioning (5 items); and, d) school functioning (5 items) [31,32]. The physical health summary score (8 items) is the same as the physical functioning scale. The psychosocial health summary score (15 items) combines the emotional, social and school functioning scales; the mean is computed as the sum of the items divided by the number of items answered in these scales. The instrument takes approximately 5 minutes to complete [32]. The Peds QL 4.0 comprises parallel child self-report and parent proxy-report formats. This scale construct consistency facilitates the evaluation of differences in HRQL across and between age groups, as well as the tracking of HRQL longitudinally. The instrument asks how much of a problem each item has been during the past 1-month period. Five-point Likert response scales are used for both the child self-report and the parent proxy-report (0 = never a problem; 1 = almost never a problem; 2 = sometimes a problem; 3 = often a problem; 4 = almost always a problem). Items are reversed; scored, and linearly transformed to a scale of 0–100 (0 = 100, 1 = 75, 2 = 50, 3 = 25, 4 = 0); higher scores indicate better HRQL. Scale scores are computed as the sum of the items divided by the number of items answered. The PedsQL 4.0 is a brief, generic measure that is widely used to assess HRQL in a range of illness groups. In clinical practice, routine clinical screening with PedsQL 4.0 has significant clinical utility [33].

### Evaluation of social support

Social support was measured with the Child and Adolescent Social Support Scale (CASSS) [34], which assesses a child’s perception of social support from four sources: teacher(s), classmates, parent(s), and close friends. Each score has 12 items. Scores were calculated by averaging the children’s frequency ratings from 1 (never) to 6 (always) for each item within the four types of support. Higher scores indicate higher perception of support. The CASSS has been well studied and has strong psychometric properties, with an alpha coefficient of 0.92 [35]. The CASSS is an appropriate measure of perceived social support for use with children and adolescents [34]. Other commonly used measures of social support for children such as the Social Skills Rating System (SSRS) [36]and the Student Self-Concept Scale (SSCS) [37]are used to measure how students feel or what they do, while the CASSS measures how children and adolescents feel about what others do to them or how others support them. The CASSS is a measure of perceived social support that is multidimensional and can advance understanding of the role of social support in the lives of children [34].

### Evaluation of disease severity

Disease severity in the present study was assessed using a binary classification of mild and severe according to specific parameters: 1) type of sickle cell disease. Sickle cell anemia (HB SS) is considered to be the most severe of the clinical syndromes, followed by sickle [*B*]-thalassemia (HB SB+) and HB SC disease [38]. The phenotype of SCD has been used as an index of disease severity in other studies [39,40]; 2) chronic transfusion therapy; 3) evidence of end organ damage with complications; 4) 12 or more medical visits for pain in the preceding 12 months; and, 5) three or more hospitalizations required per year. Participants who met none of these parameters were classified as having a mild disease. Similar indices of disease severity have been used in previous studies [41,42].

### Statistical analysis

The data analyses were performed using the statistical package for social sciences software (IBM SPSS Statistics, Version 20). Categorical data were presented in the form of number and percentage, while quantitative data were presented in the form of mean and standard deviation. Groups were compared using independent sample t-tests for quantitative parameters (95% CI). For qualitative variables, chi-square was used as a test of significance of differences among groups. Multiple linear regression was used in the analysis of the data. A *P* value of < 0.05 was considered to indicate statistical significance. The regression analysis included depression as both an independent variable (to show the predictive value of depression on HRQL) and a dependent variable (to determine the predictive value of social support and disease severity on depression).

## Results

- A total of 120 children participated in the present study. Of the 60 children with SCD (group I), 42 (70%) had HB SS, 16 (26.7%) had HB SB+, and 2 (3.3%) had HB SC disease. Forty-one children (68.3%) had severe SCD and 19 (31.7%) a mild form of the disease.
- Eight (13%) of the 60 children with SCD scored more than 12 on the CDI (mean score 14.50 ± 1.19) and on the CDI-P (mean score 14.13 ± 1.12). The clinical interviews with these eight children, using the DISC-IV, identified these children as having clinical depression (0% false-positive rate).
- As shown in Table 1, group I had significantly poorer academic performance compared with group II. No other significant differences were found between the two groups regarding age, gender, nationality, education, and family income.
- Table 2 shows that group I had significantly higher CDI and CDI-P mean scores, and significantly lower PedsQL 4.0 mean scores (self and parent report) in all domains, than children in group II.
- The multiple linear regression model used to assess the predictors of depression in children with SCD showed that a higher level of parent support was the only significant predictor associated with lower depressive states (reflected by associated lower CDI scores) in children with SCD (B = −1.79 and P = 0.01) (Table 3).
- The multiple linear regression model used to assess the predictors of quality of life in children with SCD showed that a higher level of parent support was a significant predictor associated with better quality of life, reflected by associated higher total PedsQL 4.0 self-scores in children with SCD (B = 1.89 and P = 0.02). Higher CDI scores and increased disease severity were significant predictors associated with poor quality of life, reflected by associated lower total PedsQL 4.0 self-scores in children with SCD (B = −1.70, P = 0.04 and B = −2.46, P = 0.002 respectively) (Table 4).

**Table 1:** **Demographic characteristics of children with SCD (Group I) versus healthy children (Group II)**

**Variable**	**Group I (N = 60)**		**Group II (N = 60)**		**Significance**		**CI (95%)**
	**M**	**SD**	**M**	**SD**	**t**	***p***	
Age	11.93	1.72	11.77	1.96	0.35	0.7	−0.78 – 1.12
*Range*	(10–15)		(9–15)				
	N	%	N	%	χ^2^	*p*	
Gender					0.31	0.5	
*Male*	24	40	27	45			
*Female*	36	60	33	55			
Nationality					1.22	0.2	
*Saudi*	29	48.3	23	38.3			
*Non-Saudi*	31	51.7	37	61.7			
Education					0.27	0.6	
*Elementary schoo*	34	56.6	39	65			
*Intermediate school*	26	43.4	21	35			
Academic performance					4.36	0.03*	
*Good*	44	73.3	53	88.3			
*Poor*	16	26.7	7	11.7			
Family income per month					3.3	0.1	
*<SR.3000*	9	15	7	11.6			
*SR.3000-5000*	29	48.3	21	35			
*>SR.5000*	22	36.7	32	53.4			

*P < 0.05 significant. SCD: Sickle cell disease.

**Table 2:** **CDI, CDI-P, and PedsQL 4.0 mean scores comparison between children with SCD (Group I) and healthy children (Group II)**

**Variable**	**Group I (N = 60)**		**Group II (N = 60)**		**Significance**		**CI (95%)**
	**M**	**SD**	**M**	**SD**	**t**	***p***	
CDI	9.50	2.57	7.50	1.81	3.48	0.001	0.85 – 3.15
CDI-P	8.93	3.54	7.20	1.58	2.44	0.01	0.31 – 3.16
Total score						<0.001	
*Self*	75.40	2.83	81.33	3.46	−7.25		−7.57 – -4.29
*Parent*	75.20	2.56	82.73	4.41	−8.07	<0.001	−9.40 – -5.66
Physical health						<0.001	
*Self*	77.73	2.43	82.01	2.49	−6.70		-.54 – -2.99
*Parent*	71.87	1.67	80.80	4.24	−10.71	<0.001	−10.60 – -7.26
Psycho-social health *Self*	76.37	4.51	81.63	2.52	−5.57	<0.001	−7.15 – -3.37
*Parent*	76.01	4.16	82.43	4.40	−5.80	<0.001	−8.65 – -4.21
Emotional functioning						0.03	
*Self*	76.60	9.65	80.57	2.58	−2.17		−7.62 – -0.31
*Parent*	76.83	3.79	80.56	4.09	−3.66	0.001	−5.77 – -1.69
Social functioning						<0.001	
*Self*	76.17	2.62	83.40	3.66	−8.78		−8.88 – -5.58
*Parent*	76.53	2.38	83.47	5.05	−6.79	<0.001	−8.97 – -4.89
School functioning						<0.001	
*Self*	74.33	2.27	81.01	3.04	−9.61		- 8.05 – -5.27
*Parent*	72.97	2.64	82.30	3.72	−11.19	<0.001	−11.00 – -7.66

P < 0.05 significant. SCD: Sickle cell disease, CDI: Children’s Depression Inventory, CDI-P: Children’s Depression Inventory-Parent, PedsQL: Pediatric Quality of Life.

**Table 3:** **Predictors of depression in children with SCD analyzed by multiple linear regression, with CDI scores as the dependent variable**

**Dependent variable**	**Model**	**Independent variables**	**B**	**t**	**p**
		Age	0.33	1.46	0.1
	Adjusted R^2^=	Gender	0.073	0.92	0.3
	0.37	Disease severity	0.047	0.67	0.7
CDI scores	F = 3.62	Teacher support	−0.004	−0.046	0.9
	P = 0.01*	Classmates support	−0.007	−0.081	0.9
		Parent support	−1.79	−2.73	0.01*
		Close friend support	−0.29	−1.14	0.2
		Effect of hospitalization	0.049	0.65	0.7

*P < 0.05 significant. SCD: Sickle cell disease, CDI: Children’s Depression Inventory.

**Table 4:** **QOL predictors in children with SCD analyzed by multiple linear regression; total PedsQL 4.0 self-scores as the dependent variable**

**Dependent variable**	**Model**	**Independent variables**	**B**	**t**	**p**
		Age	0.079	1.04	0.3
PedsQL total self	Adjusted R^2^=	Gender	0.072	0.85	0.7
scores	0.28	Disease severity	−2.46	−3.54	0.002*
	F = 11.95	CDI	−1.70	−2.13	0.04*
	P = 0.002	Teacher support	0.12	1.36	0.2
		Classmates support	0.10	1.13	0.2
		Parent support	1.89	2.79	0.02*
		Close friend support	0.11	1.31	0.2
		Effect of hospitalization	0.13	1.34	0.2

*P < 0.05 significant. SCD: Sickle cell disease, QOL: Quality of Life, PedsQL: Pediatric Quality of Life.

## Discussion

To the knowledge of the present authors, this is the first study that assesses the association between various types of social support, depression, and quality of life in children with SCD. Our study demonstrated that the prevalence of depression in children with SCD was 13%, a finding consistent with previous studies [9-11,43]. In a recent study, Jerrell et al [8] reported that the prevalence major depression in children and adolescents with SCD was 10%. However, in contrast to our findings, Noll et al [44] evaluated depression in a group of children aged 8–15 with SCD using the CDI, and reported that no differences were found between children with SCD and healthy children (the control group). This highlights that studies in this field continue to have conflicting results and further research is required. In addition, data for Noll et al’s study was collected at home when children were not acutely ill rather than in a hospital setting, and interviews at home may differ from those conducted in outpatient settings. In the present study, quality of life in all domains was significantly impaired (reflected in the lower PedsQL 4.0 mean self- and parent-scores in all domains) in children with SCD compared with healthy children (Table 2); a finding that is consistent with previous studies [13-15,45]. Panepinto et al [46] used the PedsQL 4.0 to determine the impact of family income and SCD on HRQL of children with SCD compared with children without the disease, and concluded that children with SCD have significantly impaired HRQL, even after considering the potential detrimental effect of family income on HRQL. In a more recent study, Dale et al [47] reported that both children with SCD and their parents rated overall HRQL and all subdomains of HRQL lower than did healthy children and their parents, a finding that matched our results. Many studies have reported that management of SCD complications may require hospitalization, therefore affecting attendance at school and normal play activities. These hospitalizations and school absences could be expected to have a negative impact on the health-related quality of life for children and adolescents with SCD [13,14,48,49].

The primary aim of the present study was to evaluate the association between disease severity, social support, and depression, and the combined and/or singular effect on HRQL in children with SCD. Our study found that disease severity and depression were associated with poor quality of life in children with SCD. Panepinto et al [45] reported that disease status was associated with impaired physical HRQL. Gil et al [50] reported that negative mood was associated with increases in same-day pain, health-care use, and reductions in school and social activity, while increases in positive mood were associated with decreases in pain, less health-care use, and more activity participation in adolescents with SCD (aged 13 to 17 years). Many researchers have examined the risk factors for developing emotional and behavioral difficulties in children; however, there has been increasing interest in the factors that promote resiliency in vulnerable children [51]. Social support is closely related to resiliency in vulnerable children, the presence of supportive parents or teachers have been identified as buffers for vulnerable children [51,52]. In our study, we examined the various types of social support that could play a role in resiliency in children with SCD (parent, teacher, classmate, and close friend support) to identify the most effective subtype of social support that is associated with resiliency in children with SCD. In addition, assessment of social support from multiple sources can advance understanding of the role of social support in the lives of children, and may enhance the development of appropriate interventions for children in need [34]. In the present study, of the various types of social support, only an increased level of parent support was significantly associated with decreased depressive symptoms and better quality of life in children with SCD (Tables 3 and 4). This means our hypothesis that increased social support is associated with decreased depressive symptoms and better quality of life is only accepted for parent support and rejected for the other types of social support (teacher, classmate, and close friend support). A support intervention study including a combination of social support plus counseling to patients with SCD found that after the intervention, patients had fewer emergency department visits and hospitalizations compared with pretreatment [53]. This indicates that support intervention is an important part of treatment of children with SCD, especially focusing on increasing the parent support that leads to decreased depressive symptoms, buffering the serious consequences of depression, and improving quality of life, which, in turn, decreases emergency department visits and hospitalizations.

## Conclusions

The present study demonstrated that a higher level of parent support was significantly associated with decreased depressive symptoms and better quality of life in children with SCD. Specific support interventions focusing on increasing parent support may be an important part of treatment of depression in children with SCD.

### Limitations

This was a cross-sectional study. Longitudinal, prospective studies may provide more reliable data on the relationship between increased parental support and decreased depressive symptoms and better quality of life. In addition, the present study did not delineate what quality/type of support might be most important (i.e., tangible, emotional, etc.), which suggests further research is necessary to provide guidance for an integrated approach to the care of these chronically ill children and adolescents.
